# Therapeutic Notes

**Published:** 1886-05-20

**Authors:** J. J. Berry

**Affiliations:** Portsmouth, N. H.


### THERAPEUTIC NOTES.

BY J. J. BERRY, M. D., OF PORTSMOUTH, N. H.

The following items which are taken from my note book have been of such use to me that I am led to think they may furnish useful hints to others as well. Few of them are original, but have been derived from all possible sources and have been utilized by me to a greater or less extent.

Tonics should be administered with certain alteratives when given for long periods of time ; for instance, quinine with mercury; cod-liver oil with iodide of potash; and iron with the various salines.

Emetics are not given enough. Effects upon the liver and intestinal tract are often more obtainable in this way than by any other.

The diarrhœa of phthisis is usually relieved by a pill of g. terebinth, cupri sulph. and opium.

The best treatment of engorgement of bronchial mucous membrane is to keep the bowels open.

The best respiratory stimulants are ammonia, atropia, strychnia, ipecac and squills.

*Expectorant.*—Eth. sulph., gtts. v–v, every three or four hours.

*Pruritus* (general).—Potass. brom., grs. x; potass. liq. gtts. x; three to four times daily.

*Lumbago.*—Potass. cit., grs. xx ter die with hypodermics of morphine and atropia.

Salines act better after physiological doses of calomel.

The action of quinine is increased by half drachm doses of bitrart. of potash.

Belladonna or bromide of potash often prevents the disagreeable effects of iodide of potash.

Alcohol helps the organism to tolerate large doses of quinine.

The addition of alcohol to croton oil renders the latter less powerful but none the less effective.

*Chorea.*—F. E. cimicifuga in gradually increased doses.

Columbo checks colliquative diarrhœa and relieves irritability.

Senega for the bronchitis and emphysema of old people Balsams act best in chronic diseases of the bronchial mucous membrane.

The secretion of urea is increased by sod. benzoat, colchicum and hydrarg bichlorid, as well as by the salts of potash.

Salines have an increased cathartic effect if given in a concentrated form, withholding all liquids.

Iodine is one of the best anti-suppurative remedies. It has been used with succes in typhoid fever and pneumonia.

Calomel, one half grain, is said to relieve dryness of tongue in six hours.

*Tonsilitis.*—Local applications of bicarbonate of soda.

The cough of phthisis is often relieved by remedies which act upon the liver.

Counter irritation of the neck, over course of pneumogastric nerves will often relieve asthma.

Arsenic is one of the best tonics in chlorosis and the anæmia of women.

A saturated solution of sod. bicarb. best relieves pain of cautery burns.

Acids best relieve phthisical dyspepsia.

Chloroform increases the secretions.

Chloral hydrate or chloroform water in painful affections of the stomach.

Grain doses of ipecac act as a hepatic stimulant.

The pains of spinal irritation are often relieved by arsenic.

Vaseline is much used for conjunctivitis.

Caffeine slows the action of the heart, increases the amount of urea and decreases the amount of albumen excreted.

Salicin will often relieve dysmenorrhœa. It is of great value in tonsillitis and muscular pain.

*Phlegmasia dolens.*—Opium with alterative doses of calomel.

*Epilepsy.*—A hypodermic of morphine will often abort a paroxysm.

*Sick-headache.*—A hot foot bath with a fifteen grain dose of chloral.

*Hoarseness.*—Tr. guaiac am. ten drops on sugar every half hour. Steam inhalations.

*Facial Neuralgia.*—Tr. gelsem., three drops every half hour. Salicylate of soda.

*Weak Heart.*—Tr. stramonium and tr. digitalis, of each ten drops, three times daily.

*Lead Paralysis.*—Large doses of strychnia.

*Diarrhœa (in teething children).*—Infus. camomile.—Columbo.

*Regurgitation (of Infants).*—Calomel gr. j; aq. Oj; teaspoonful every fifteen minutes.

*Eructation, Pyrosis and Fermentation.*—Ac. carbol, gr. i–ii in mint water.

Inhalations of steam impregnated with oil of peppermint is almost a specific in relieving painful affections of the throat.

Calomel is one of the very best remedies in many of the acute diseases of children ; the latter is so very tolerant of the drug that it is almost impossible to salivate with laxative doses. Its antipyretic effects and its sedative action upon the intestinal tract is appreciated only by those who have used it.

The value of carbonate of ammonia in the respiratory diseases of children is not fully recognized. It should be given in large doses well diluted with milk.

Ovarian pain is best relieved by belladonna, arsenic, velerianate of zinc and carbonate of iron.

Muriate of ammonia relieves congestion of the pelvic viscera.

Permanganate of potash in two grain doses is an excellent emmenagogue for strumous subjects.—*New England Med. Monthly.*

